# Unusual combined thymic mucoepidermoid carcinoma and thymoma: a case report and review of literature

**DOI:** 10.1186/1746-1596-9-8

**Published:** 2014-01-20

**Authors:** Shi-gang Wu, Yang Li, Bin Li, Xiao-ying Tian, Zhi Li

**Affiliations:** 1 Department of Pathology, The First Affiliated Hospital, Sun Yat-sen University, 58, Zhongshan Road II, Guangzhou 510080, China; 2 Department of Pathology, Qingyuan People’s Hospital B24, Yinquan Road, Qingcheng District, Qingyuan City 511518, China; 3 School of Chinese Medicine, Hong Kong Baptist University 7, Baptist University Road, Kowloon Tong, Hong Kong, China

**Keywords:** Thymic neoplasm, Combined thymic epithelial tumor, Mucoepidermoid carcinoma, Thymoma, Differential diagnosis

## Abstract

**Background:**

In rare condition, combined thymic epithelial tumors showing either type A or type B thymomas areas combined with thymic carcinoma components may occur in thymus. Mucoepidermoid carcinoma (MEC) of the thymus is rare in thymic carcinoma, and so far there is no report to describe a combined epithelial tumor of thymus with MEC component. We report an unusual case of combined thymic MEC/type B2 thymoma in a middle-aged male occurring in a mass of anterior mediastinum. Case report: A 51-year-old Chinese male patient presented with a 6-month history of right ptosis and progressive muscle weakness. Computed tomography (CT) examination revealed a solitary, well-circumscribed mass was in the anterior mediastinum with mild heterogeneous enhancement. Histologically, the mass contained two separated components and displayed typically histological features of low-grade MEC and type B2 thymoma, respectively. There was no gradual transition of these two components observed in mass, and no enlarged lymph node was found in the surrounding tissues. A diagnosis of combined thymic MEC/type B2 thymoma was made. The patient received thymectomy to resect the mass totally. After surgery, chemotherapy with regiments of cisplatin and mitomycin, and radiotherapy of the main tumor bed were performed on the patient. There was no evidence of tumor recurrence during the period of 12 months follow-up.

**Conclusion:**

To our best knowledge, this is the first report of combined thymic epithelial tumor with MEC component. Although this tumor is rare, the diagnosis of a thymic MEC should be taken into consideration when a combined epithelial tumor is occasionally encountered in thymus.

**Virtual slides:**

The virtual slide(s) for this article can be found here: http://www.diagnosticpathology.diagnomx.eu/vs/9721397571157894

## Background

Primary thymic carcinoma is a rare tumor of the anterior mediastinum. Mucoepidermoid carcinoma (MEC) of the thymus is rare in thymic caircinoma, and comprises approximately 2% of published thymic carcinoma [1,2]. Up to date, no more than 30 cases of thymic MEC have been described in the literature [3-15]. In rare condition, combined thymic epithelial tumors showing either type A or type B thymomas areas combined with thymic carcinoma components may occur primarily in thymus, and are exceptionally rare (<1%) [16]. Among the combined thymic epithelial tumors, over 80% of tumors have typical B2 and B3 differentiation. The carcinoma component in the most case is a squamous cell carcinoma. Lymphoepithelioma-like, sarcomatoid/anaplastic or undifferentiated carcinomas are uncommon. However, to our knowledge, so far there is no report to describe a coexistence of thymoma and primary MEC within the same mediastinal tumor in English literatures. Herein we report a middle-aged male patient with MEC combined with type B2 thymoma in the same nodule of anterior mediastinum. Histopathological findings and imaging features, as well as outcome and differential diagnosis are to be discussed.

## Case presentation

### Patient and clinical management

**Figure 1:**
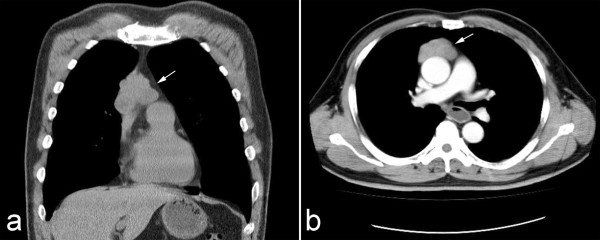
**Preoperative chest radiology of lesion. (a)** Chest CT image showed a well-defined mass in right anterior mediastinum (white arrow). The tumor was observed to adhere to the wall of great vessel. **(b)** The contrast enhanced CT scan showed the solid mass with mild heterogeneous enhancement adhered, but not invaded the wall of great vessel.

A 51-year-old Chinese male presented with a 6-month history of right ptosis and progressive muscle weakness. The patient had been diagnosed as myasthenia gravis at local hospital 6 months before, but for unknown reasons, he failed to receive workup and management at that time. Before one day the patient was admitted to our hospital, he suffered with a sudden onset of dysarthria. Therefore, the patient referred to our hospital for further examination and treatment. Physical examination showed right-sided partial ptosis. Diplopia was noticed on right lateral gaze due to right lateral rectus weakness. The patient did not have dysphagia or dyspnoea. But he was having generalized weakness in all his extremities, both proximal and distal, with marked diurnal variation in the form of more weakness during the evening. A confirmatory electrophysiological study with repetitive nerve stimulation showed decrement in amplitude of action potentials with further reduction post exercise and recovery after 15 minutes. Acetylcholine receptor (AChR) binding antibodies were markedly elevated. However, other routine laboratory test, including blood count, differential, liver and renal function, were within the normal range. A chest computed tomography (CT) scan demonstrated a solid mass, measuring 5.4 × 3.7 × 2.6 cm, with focal heterogeneously enhancement, in the right anterior mediastinum. The tumor was observed to adhere to the wall of aorta but did not infiltrate it. Minimal amount of right pleural effusion and pericardial effusion were associated, but neither enlarged lymph node nor remarkable image finding was noted in the lung parenchyma (Figure 1). A CT guided fine needle biopsy of the anterior mediastinal mass was performed and pathological examination showed predominantly epithelial neoplastic cells with pan-cytokeratin (AE1/AE3) positive in a lymphoid component background suggesting a thymoma. Therefore, a preoperative diagnosis was thymoma and the tumor was totally resected. At surgery, the main tumor located in the right lobe of the thymus was found to have adhered to the periaortic tissues, but no invasion of the wall of aorta was observed. Thymectomy and resection of the adherent tissues were performed.

### Hisopathological findings

**Figure 2:**
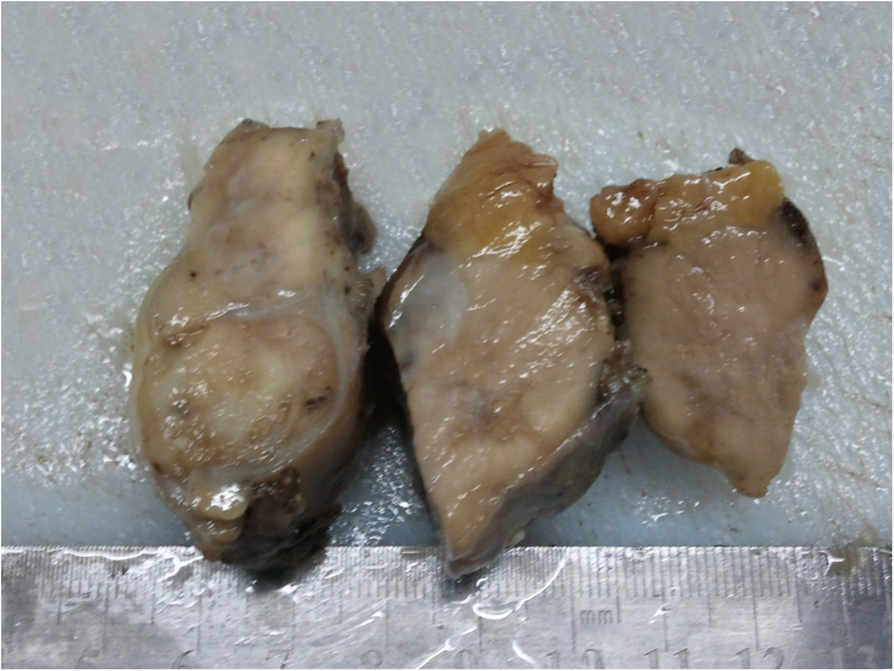
**Postoperative gross examination of mass.** On macroscopical examination, the lesion was gray-red solitary nodular mass without gross necrosis, haemorrhage and calcification. There was no fibrous capsule round the mass.

**Figure 3:**
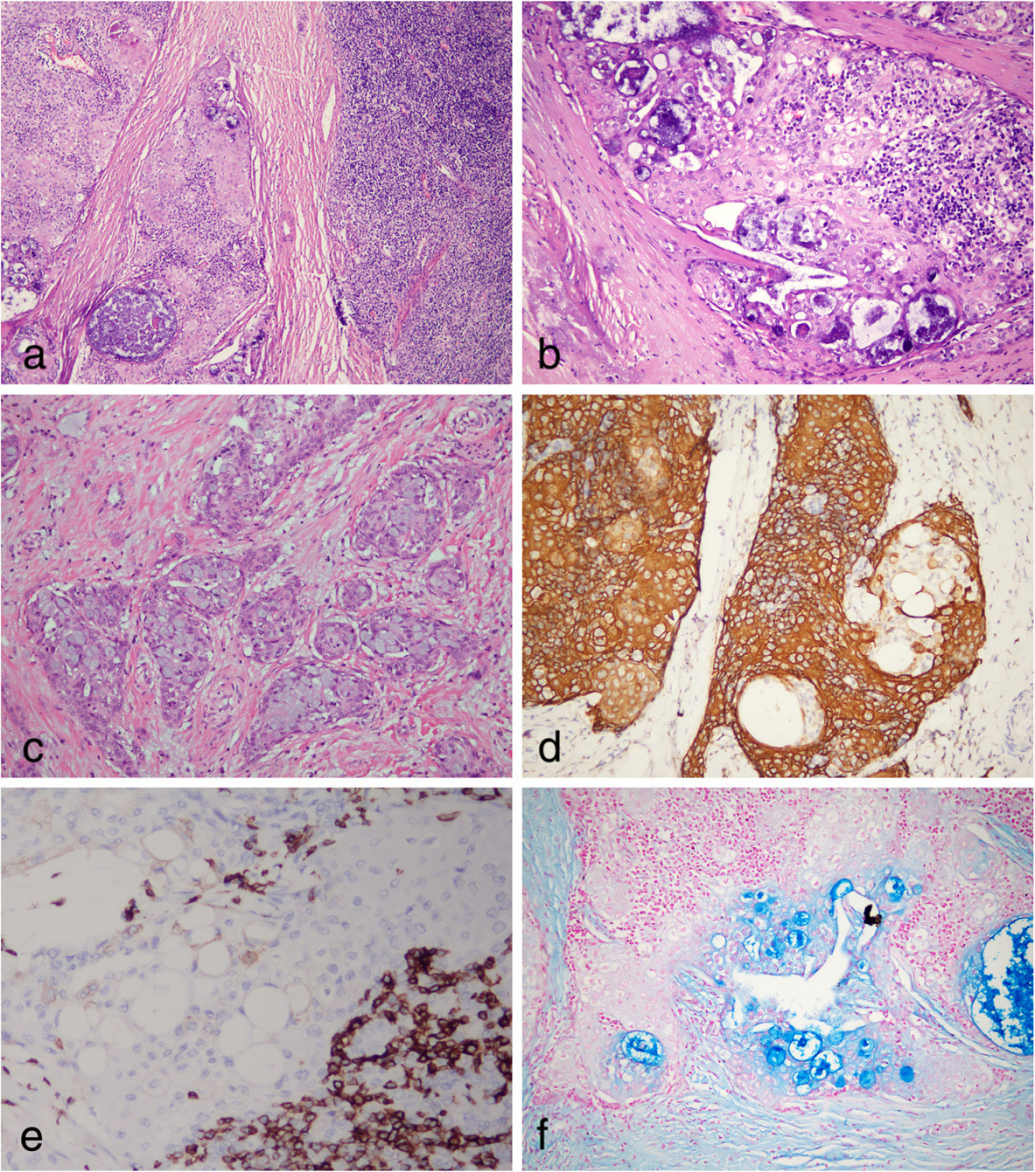
**Postoperative photomicrographs of lesion in MEC component. (a)** The tumor was composed of nests of epidermoid cells with mucin-producing areas (left side of the figure) and lymphocyte-rich areas (right side of figure). They represented the MEC component and thymoma component, respectively. **(b)** High magnification showed the mucin-producing cells were surrounded by epidermoid cells in MEC component. **(c)** Invasive nests of well-differentiated epidermoid cells, mucin-producing and intermediate cells were observed in MEC areas. Bands of fibrous connective tissue were observed in the tumor. **(d)** The epidermoid cells were observed to be positive for CK5/6, but the mucin-producing cells were CK5/6-negative. **(e)** MEC component was negative for CD5. **(f)** The Alcian blue-positive material was seen in lumen of gland structure and the mucin-producing cells within the nest of epidermoid cells. (**a**, H & E staining with original magnification of 200 ×; **b**-**c**, H & E staining with original magnification of 400 ×; **d**-**e**, immunohistochemical staining with original magnification of 400 ×; **f**, Alcian blue staining with original magnification × 400).

**Figure 4:**
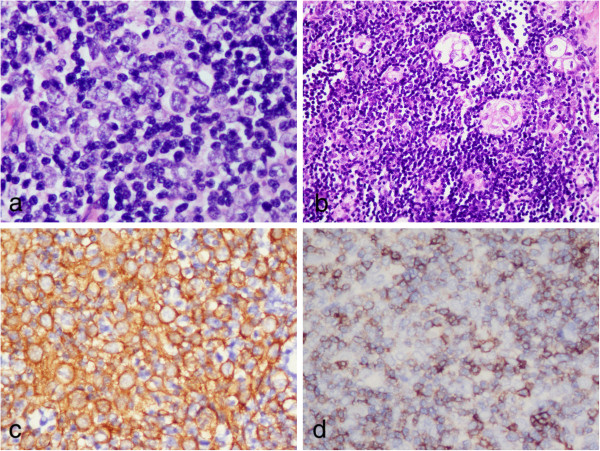
**Postoperative photomicrographs of lesion in thymoma component. (a)** The neoplastic cells were large and polygonal with large nuclei and prominent central nucleoli in the lymphocyte-rich background. **(b)** Some small epidermoid foci resembling abortive Hassall’s corpuscles could be observed among the neoplastic epithelial cells, but no mucin-producing cells were found in these epidermoid foci. In these areas, the neoplastic cells were diffusely positive for CK19 **(c)**, but negative for CD5 **(d)**. (**a-b**, H & E staining with original magnification of 400 ×; **c-d**, immunohistochemical staining with original magnification of 400 ×).

Macroscopical examination revealed a gray-red solitary nodular mass, and measured 5.0 × 3.5 × 2.0 cm. The mass was well-circumscribed, but there was no fibrous capsule around the mass and no cystic cavity observed in the mass (Figure 2). Under the microscopical examination, part of nodular mass was composed of solid and invasive nests of well-differentiated epidermoid cells with desmoplastic stroma. The mucin-producing and intermediate cells were also observed in the tumor. These tumor cells were intermingled or intimately mixed with epidermoid cells. Mucin-producing cells were cuboidal, columnar, or goblet-like with bland nuclear morphology. Bands of fibrous connective tissue were observed among the neoplastic elements. There was no extensive necrosis and neural invasion. Mitotic figures were infrequent and there was no remarkable cellular pleomorphism. Immunohistochemically, the epidermoid cells of the tumor were positive to pan-CK (AE1/AE3), CK5/6, CK7 and p63, but negative to CD5. Mucin-producing cells were negative to CK5/6 and p63. Alcian blue staining revealed mucin in the cytoplasm of the mucin-producing cells (Figure 3). This part of tumor was diagnosed as low-grade MEC according to the histopathological criteria of WHO classification [17]. Interestingly, however, type B2 thymoma could be observed in other area of the same nodular mass. In this area, neoplastic cells were large and polygonal with large nuclei and prominent central nucleoli. The neoplastic cells formed delicate lose network, and small epidermoid foci resembling abortive Hassall’s corpuscles could be observed among the neoplastic epithelial cells, but no mucin-producing cells were found in these epidermoid foci. In these areas, the neoplastic cells were outnumbered by non-neoplastic lymphocytes. By immunohistochemical staining, the neoplastic cells revealed the positivity for pan-CK (AE1/AE3), CK5/6, CK19 and p63, as well as negativity for CD5 and CD117 (Figure 4). The field of MEC and type B2 thymoma was distinct in the mass, there was no gradual transition of these two parts observed in mass although they were close to each other in some areas. Based on above findings, a final histological diagnosis of primary combined type B2 thymoma/MEC of thymus was made, and the final staging of this tumor was stage II (Masaoka staging system).

The postoperative phase was uneventful and the dysarthria resolved. After diagnosis, the patient was started on pyridostigmine with a remarkable improvement in weakness, diplopia and ptosis. Chemotherapy with regiments of cisplatin and mitomycin, and radiotherapy of the main tumor bed were performed on the patient. Since there was a possibility of tumor metastasis to another anatomical location, the patient was referred to a whole body positron emission tomography (PET)/CT study to search for the potentially secondary tumor, but no abnormality was found. The patient was on regular follow-up for 12 months after discharging from hospital. He had remained asymptomatic, and there was no evidence of tumor recurrence during the period of postoperative follow-up.

## Discussion

Combined thymic epithelial tumors are rare and characterized by at least two distinct areas each corresponding to one of the histological thymoma and thymic carcinoma types [16]. The etiology of these tumors remains enigmatic. Some genetic studies suggested that combined thymic epithelial tumors could arise by dedifferentiation of thymoma/thymic carcinoma or by biphasic differentiation of a multipotential thymic epithelial precursor. However, the concept of tumor collision awaits genetic evidence [3]. Clinically, almost all reported cases of combined thymic tumors were observed in the anterior mediastinum, and there were no differences in the clinical manifestations of combined tumors as compared to the individual component. Myasthenia gravis (MG) is by far the most common paraneoplastic manifestation. Histologically, over 80% of combined tumors have type B2 and B3 component. But rare cases of combined type AB thymoma or spindle cell (type A) thymoma with thymic carcinoma has also been described [18,19]. The carcinoma component in the most cases is squamous cell carcinoma. Lymphoepithelioma-like, sarcomatoid/anaplastic or undifferentiated carcinomas are uncommon. In the present case, the typical MEC and type B2 thymoma could be identified in different areas of the same tumor. The patient showed a mass in anterior mediastinum with typical manifestations of myasthenia gravis. These findings were consistent with the diagnostic criteria of combined type B2 thymoma/MEC of thymus. To our best knowledge, so far there is no report to describe a coexistence of thymoma and primary MEC within thymic tumor. Our case is the first case of combined thymic epithelial tumor with MEC component.

MEC is a relatively common neoplasm of the salivary glands, which rarely arises in other sites, including esophagus, anal canal, skin of the breast, lachrymal sac, thyroid gland, or uterine cervix [20-23]. Primary thymic MEC is rare. It was first described by Snover DC and his colleagues in 1982 [3]. Since then, no more than 30 cases have been reported in the English literature [4-15]. Despite its distinct histological morphology, the pathogenesis of MEC of thymus is still unknown. It has been suggested that thymic MECs might arise from thymic epithelium because, in some cases, a transition between tumor cells and benign cyst-lining epithelium and the finding of residual non-neoplastic thymic parenchyma within the walls of the cysts has been reported in some cases [7]. However, pluripotent epithelial stem cells of endodermal origin have been also postulated in the pathogenesis of MEC of the thymus by some authors [3]. A strong association between MEC and t (11; 19) (q21; p13) has been observed in non-thymic anatomical sites [24,25]. Recent study has demonstrated that MAML2 rearrangement, a member of the Master Mind Like gene family on chromosome 11q21, is harbored specifically in thymic MEC similar to MEC in other anatomical sites, suggesting thymic MEC is not only histologically but also biologically related to non-thymic cases of MEC [15]. It is necessary for the further study to clarify if MAML2 rearrangement presents in the rare MEC component of combined thymic epithelial tumors.

The establishment of a preoperative diagnosis of thymic MEC is difficult because of the rarity of this tumor and the fact that there are no specific landmarks in the radiologic examinations. Therefore, percutaneous biopsy is needed for this tumor to obtain a definite diagnosis preoperatively [14]. In the current study, a CT guided fine needle biopsy of the anterior mediastinal mass was performed. However, only type B2 thymoma was observed in the biopsy because it was difficult for pathologists to provide a precise diagnosis from a few very small tumor tissues in rare combined thymic tumor. Under these extremely rare conditions, sufficient tissue from different parts of the lesion and thoroughly histological inspection are necessary for accurate diagnosis. Even if a diagnosis of thymic MEC is confirmed by biopsy examination, the possibility of a metastasis from primary MEC occurring in common sites should also be excluded. Thoroughly body examination and a whole body PET/CT study are useful to find the potentially primary tumor. In our case, no primary tumor of MEC was found by whole body PET/CT study.

Histologically, MEC in thymus and other anatomical sites are characterized by squamoid (epidermoid), mucin-producing and cells of intermediate type with varying proportion and architectural configuration in and between tumors [26]. The presence of mucin-producing cells in the tumor is the key diagnostic clue for MEC. However, the presence of mucinous differentiation in a thymic neoplasm is not uncommon. Mucinous epithelium can be occasionally noted in the normal human thymus and in thymomas [27]. It more frequently occurs in the thymus of dogs and other animals, and reflects the potential of the thymic epithelium to differentiate along multiple cell lines [4]. Thus, thymic MEC might be confused by thymomas with mucinous differentiation. However, the latter lacks the intermediate type cells and invasive nests of epidermoid cells with desmoplastic stroma. Thymic MEC is sometimes misdiagnosed as squamous cell carcinoma of thymus as the tumor presents predominantly nests of invasive epidermoid cells with inconspicuous mucin-producing cells component. Since the MEC is rare in thymus, these morphological features might be erroneously interpreted squamous cell carcinoma by those who were not familiar with this condition. However, mucin-producing cell is absent in the squamous cell carcinoma, which can be demonstrated in majority of tumor by Alcian blue and diastase-PAS staining. Sufficient tissue from different parts of the tumor and thorough inspection to find the mucin-producing cells will facilitate the precise diagnosis of MEC. More importantly, thymic squamous cell carcinomas are immunoreactive to CD5, which are quite useful to distinguish it from thymic MEC and other non-thymic origin squamous cell carcinoma. Like most of reported cases, the MEC component of our case also presented the immunohistochemical negativity to CD5, supporting the diagnosis of MEC rather than squamous cell carcinoma. However, only one previously reported case of thymic MEC showed tumor cells were positive to CD5 [14]. It is not clear whether this immunoreaction represents a specifically diagnostic marker or an aberrant expression. More cases of primary thymic MEC should be needed to confirm this immunophenotype. In addition, multilocular cystic structures may be frequently observed in thymic MEC, the differential diagnosis of cystic masses in anterior mediastinum, even rare ectopic pancreatic pseudocyst, should be included [28].

Available data on combined thymic epithelial tumors suggest that the most aggressive component of tumor determines the clinical outcome. As to thymic MEC, high-grade tumors have been demonstrated more invasive and shown stronger tendency for metastasis compared with low-grade tumors [18,19]. Among the cases reported with known pathological grades and clinical courses, mortality was limited to the high-grade type of MEC. In the present case, the MEC component was low-grade type according to the WHO grading criteria because the tumor lacked the histological features of neural invasion, cystic formation, necrosis and active mitotic figures [17]. Type B2 thymoma is a tumor of moderate malignancy with invasiveness and recurrence, even after complete resection. Recent study has demonstrated that the overexpression of c-Jun, p73 and Caspase-9 in thymic epithelial tumors is closely related with the pathogenesis and biological behavior of the neoplasms [29]. To date, there are no established therapeutic regimens for thymic MEC because of the rarity of this tumor. The published experience with chemotherapy for MEC has been limited, but the tumors have been suggested to be chemosensitive [30]. Most regimens for thymic carcinoma are similar to those for thymoma and include cisplatin [31,32]. Significant beneficial effect of cisplatin-based combination chemotherapy for inoperable thymic carcinomas has been suggested in several studies [31-33]. In the present case, there was no evidence of tumor recurrence during the period of postoperative follow-up. We presume that low-grade of tumor and chemotherapy with cisplatin regimen might be associated with favorable results. Of course, a longer follow-up period and laboratory examinations are needed to inspect the long term prognosis of our patient.

## Conclusions

In summary, we herein first reported a rare case of combined thymic MEC/thymoma in an anterior mediastinal mass. The mass contained two separated components and displayed typically histological features of low-grade MEC and type B2 thymoma, respectively. The precise mechanism of coexistence of MEC and thymoma in the same nodule remains unknown, and longer period of follow-up and more case investigation are necessary to better clarify the biological characteristics and clinical outcomes of this unusual tumor. Although this tumor is rare, the diagnosis of a thymic MEC should be taken into consideration when a combined epithelial tumor is occasionally encountered in thymus.

## Consent

Written informed consent was obtained from the patient for publication of this case report and any accompanying images. A copy of the written consent is available for review by the Editor-in-Chief of this journal.

## Competing interests

The authors declare that they have no competing interests.

## Authors’ contributions

SGW and YL made contributions to acquisition of clinical data, and analysis of the histological features by H & E staining and immunoassays. They are joint first co-authors and made an equal contribution to this work. BL carries on the immunohistochemical and special staining. XYT drafted the manuscript. ZL revised manuscript critically for important intellectual content and had given final approval of the version to be published. All authors read and approved the final manuscript.
